# A tool kit for rapid cloning and expression of recombinant antibodies

**DOI:** 10.1038/srep05885

**Published:** 2014-07-30

**Authors:** Tihomir S. Dodev, Panagiotis Karagiannis, Amy E. Gilbert, Debra H. Josephs, Holly Bowen, Louisa K. James, Heather J. Bax, Rebecca Beavil, Marie O. Pang, Hannah J. Gould, Sophia N. Karagiannis, Andrew J. Beavil

**Affiliations:** 1 NIHR Biomedical Research Centre at Guy's and St. Thomas's Hospitals and King's College London, London, UK; 2 St. John's Institute of Dermatology, Division of Genetics and Molecular Medicine, King's College London School of Medicine, King's College London, London SE1 9RT, UK; 3 Division of Cancer Studies, King's College London, 3rd Floor Bermondsey Wing, Guy's Hospital, London SE1 9RT, UK; 4 Randall Division of Cell and Molecular Biophysics, King's College London, Guy's Campus, London SE1 1UL, UK

## Abstract

Over the last four decades, molecular cloning has evolved tremendously. Efficient products allowing assembly of multiple DNA fragments have become available. However, cost-effective tools for engineering antibodies of different specificities, isotypes and species are still needed for many research and clinical applications in academia. Here, we report a method for one-step assembly of antibody heavy- and light-chain DNAs into a single mammalian expression vector, starting from DNAs encoding the desired variable and constant regions, which allows antibodies of different isotypes and specificity to be rapidly generated. As a proof of principle we have cloned, expressed and characterized functional recombinant tumor-associated antigen-specific chimeric IgE/κ and IgG_1_/κ, as well as recombinant grass pollen allergen Phl p 7 specific fully human IgE/λ and IgG_4_/λ antibodies. This method utilizing the antibody expression vectors, available at Addgene, has many applications, including the potential to support simultaneous processing of antibody panels, to facilitate mechanistic studies of antigen-antibody interactions and to conduct early evaluations of antibody functions.

In the last 30 years, antibody-based immunotherapies have been applied to treat a variety of diseases^1^ and there is a constant development of novel antibodies^2^,^3^,^4^. This has required the selection of recombinant monoclonal antibodies (mAbs) with high affinity for the appropriate epitopes on the target antigen and other desirable characteristics, such as the antibody isotype and effector functions. Large cDNA libraries of the variable (V) gene segments of antibodies are frequently generated in the study of immune disorders that involve B-cells, such as autoimmune diseases^5^, allergy and asthma^6^,^7^,^8^,^9^,^10^,^11^,^12^ or cancer^13^,^14^,^15^,^16^,^17^. While this provides an insight at the genetic level, the antigen binding and effector functions may only be elucidated from the expressed antibody. Similarly, large libraries of scFv may be generated using phage display experiments and screened to identify antigen specificity^18^, and would benefit from a method of rapid reformatting as full-length antibodies, in order to evaluate their biological function.

Conventional methods for recombinant expression of whole antibodies of interest typically require the establishment of cell lines derived from CHO, mouse myeloma, or PER.C6 cells^19^,^20^,^21^,^22^,^23^,^24^. This tends to be a lengthy, low efficiency process involving extensive selection and screening and is consequently unfavourable for expressing large numbers of antibodies for functional studies, as may be required after generating a variable gene library.

Matched Ig heavy- and light-chain V-regions of desired specificity have been amplified from single B cell-derived cDNA and linked to the desired Ig constant (C) region in separate vectors encoding either the heavy- or light-chain DNA. Transient transfection of these vectors in mammalian HEK293 cells has enabled the rapid production of recombinant mAbs^12^,^25^,^26^,^27^,^28^. Although these facilitate expression of a large number of mAbs in a short time, in our experience, the transient co-transfection of separate vectors doubles the DNA preparation work, requires maxi or mega plasmid purifications and large amount of transfection reagent for the production of milligrams of antibody, which is a costly, labor- and time-intensive scale-up process. This limitation has been addressed by the generation of vectors hosting a dual antibody expression cassette with a selection marker^29^,^30^,^31^. However, all these antibody cloning systems rely on restriction enzyme- and ligation-dependent cloning methods, which adds an additional cost, prevents high-throughput analysis of antibody candidates, and may sometimes be impractical due to the lack of compatible restriction sites.

A suitable high-throughput antibody cloning method would enable reliable assembly of antibody heavy and light chains in a single vector via a cheap, rapid, and convenient platform. An alternative to the conventional cloning methods, the Polymerase Incomplete Primer Extension (PIPE) method, has been shown to be highly efficient, cost-effective and capable of supporting the high-throughput cloning of thousands of genes in parallel in a short period of time^32^. Therefore, utilizing the PIPE cloning method, we sought to develop an improved flexible system for cloning of antibodies of any isotype and specificity. We aimed to minimize the cost and time required to clone antibody heavy and light chains into a single mammalian expression vector for stable transfection of a suitable cell line, allowing an easily scalable production process and delivering sufficient material for pre-clinical studies.

In this study we describe an efficient and cost-effective method for antibody cloning that facilitated the seamless exchange of antibody variable and constant domains to produce fully functional recombinant antibodies. As a proof-of-principle, we engineered tumor-associated antigen chondroitin sulfate proteoglycan 4 (CSPG4) specific chimeric IgE/κ and IgG_1_/κ and grass pollen allergen Phl p 7 specific human IgE/λ and IgG_4_/λ antibody isotypes. The choice of these antibodies was dictated by the search for antibodies with potential applications for the diagnosis or immunotherapy of melanoma and allergy, respectively.

## Results

### Antibody Cloning by PIPE

**Figure 1:**
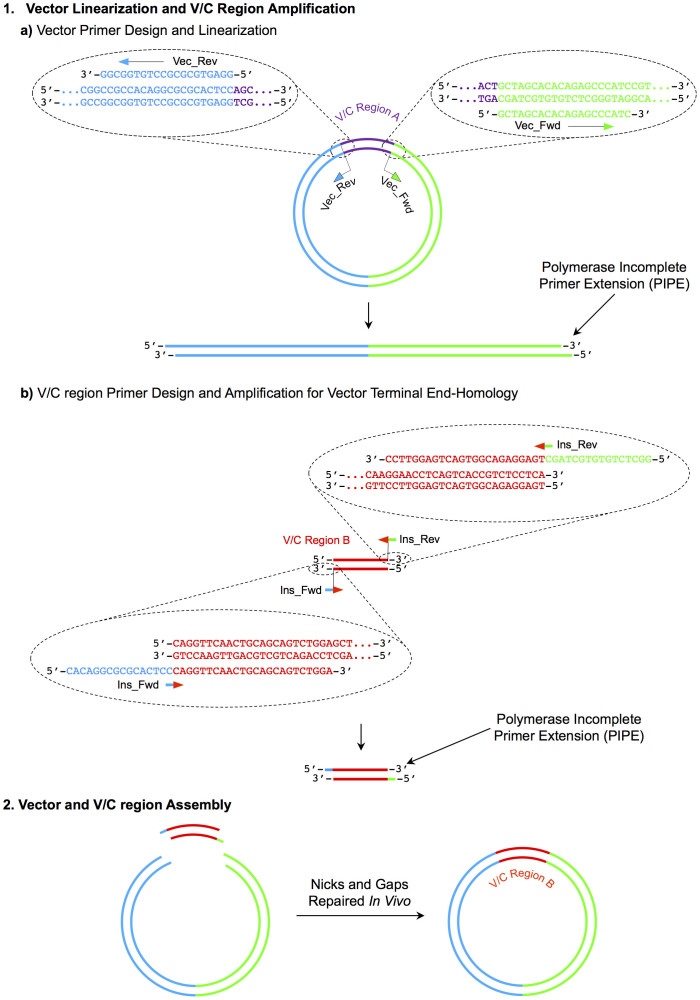
Schematic representation of primer design for PIPE cloning. 1 A) A set of primers flanking the existing V/C antibody region are designed for vector linearization. 1 B) Simultaneously, the desired V/C antibody region is amplified by another set of 3′ V/C region specific, 5′ vector-end homologous primers. 2) The incomplete extension products anneal directionally across the complementary sequences encoded in the primers and nicks and gaps are repaired *in vivo* after transformation.

We adopted the PIPE cloning method for construction of antibody expression vectors and swapping of variable/constant regions. The antibody cloning method represents a simple two-step protocol with complete design flexibility (Figure 1). In the first step, a pair of 40 bp vector-specific primers is used for PCR vector linearization and generation of single-stranded 5′-ends by PIPE (Figure 1, Step 1a). Simultaneously, another pair of 40 bp primers with 5′-vector-end overlapping sequences is used for insert PCR amplification, generating single-stranded vector-end homologous products by PIPE (Figure 1, Step 1b). In the second step, the unpurified PIPE products are mixed and the single-stranded overlapping sequences anneal and assemble as a complete vector (Figure 1, Step 2).

Following the steps outlined in Figure 1, we designed the construction of a dual antibody expression cassette into pVITRO1 mammalian expression vector (see Supplementary Fig. S1 online). PCR linearization of pVITRO1 at Multiple Cloning Site 2 (MCS2) allowed the insertion of a heavy chain expression cassette under the action of a SV40 enhancer. In a subsequent PIPE cloning reaction, a sequence verified clone pVITRO1-IgE (containing the human Epsilon heavy chain expression cassette at MCS2) was used as a template for PCR vector linearization at MCS1. This allowed the insertion of a human light chain (Lambda or Kappa) expression cassette under the action of a human CMV enhancer. Transformants were sequenced across the annealing junctions to confirm that the dual antibody expression vectors pVITRO1-IgE/λ and pVITRO1-IgE/κ were successfully assembled (see Supplementary Fig. S1 online).

**Figure 2:**
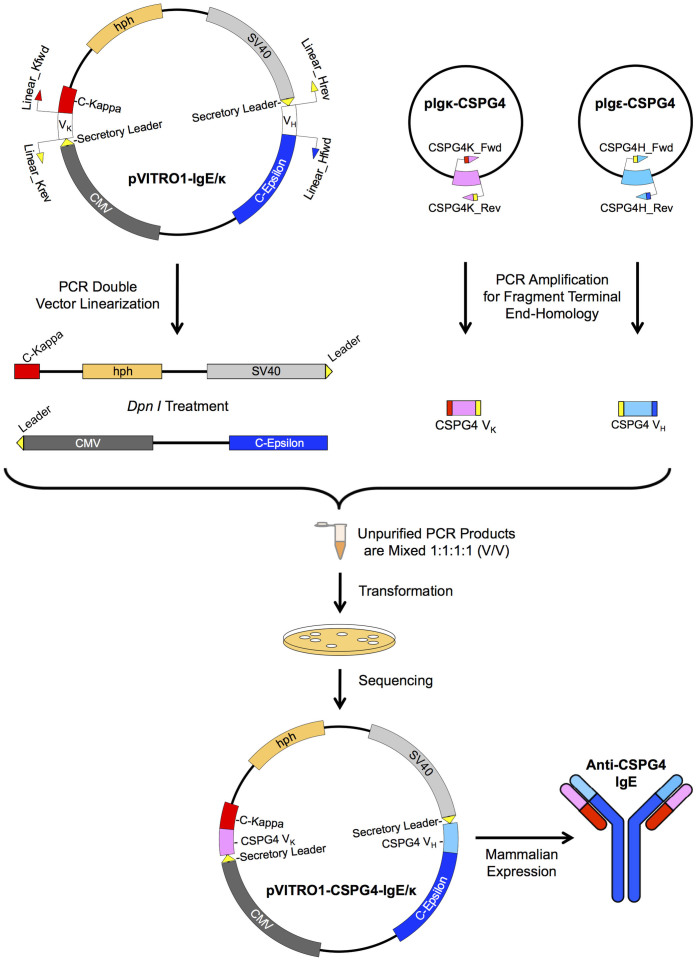
Schematic representation of PIPE cloning strategy for swapping antibody variable regions. pVITRO1-IgE/κ vector is PCR linearized by V_H_ and V_K_ flanking primer pairs in two independent PCR reactions, resulting in two vector fragments subsequently treated with *Dpn*I. Simultaneously, the CSPG4 specific V_H_ and V_K_ are PCR amplified for generation of vector fragment terminal end-homology. The *Dpn*I-treated vector fragments are mixed with unpurified V_H_ and V_K_, the single-stranded DNA fragments anneal directionally across the complementary sequences and nicks and gaps are repaired *in vivo* after transformation, generating pVITRO1-CSPG4-IgE/κ expression vector.

Having successfully utilised the PIPE cloning method for vector construction we aimed to swap antibody variable regions. This process involved exchanging existing V_H_ and V_K_ regions in pVITRO1-IgE/κ, without affecting the Cε and Cκ. To achieve this, four primer pairs were designed. The first two vector-specific primer pairs, flanking the existing V_H_ and V_K_, were used for amplification of two vector fragments, subsequently treated by *Dpn*I to destroy the *E. coli*-derived PCR template (Figure 2).

Agarose gel electrophoresis analysis of the PCR amplified vector fragments showed clear DNA products with no unspecific amplifications (see Supplementary Fig. S2 online) and sequencing identified no mutations. The second two primer pairs, with 5′-vector-fragment-end overlapping sequences, were used for amplification of the incoming CSPG4-specific V_H_ and V_K_ (see Supplementary Figure S2 online). The four unpurified PCR products were simply mixed together (v/v) and transformed into chemically competent *E. coli* cells (NEB 10-beta). DNA sequencing of a single colony confirmed the correct annealing of the vector fragments with the CSPG4-specific V_H_ and V_K_, assembling a complete pVITRO1-CSPG4-IgE/κ vector, coding for the expression of chimeric anti-CSPG4 IgE antibody (Figure 2). Similarly, a pVITRO1-102.1F10-IgE/λ vector coding for the expression of anti-Phl p 7 IgE was generated, demonstrating the system's simplicity to exchange variable-region domains within the same antibody isotype. Furthermore, a high-throughput antibody cloning method requires a minimum number of colonies to be picked and tested for correct assembly. Therefore, the efficiency of the PIPE cloning method to swap variable regions was compared against the Gibson Assembly (NEB) and GeneArt® Seamless Cloning (Life Technologies) kits, following the manufacturer's instructions to represent optimized conditions for these methods. Sequencing of plasmids, purified from single colonies, over the annealing junctions showed comparable number of colonies with correctly assembled vectors by PIPE and the two commercially available kits (see Supplementary Table S1 online). In the PIPE cloning reaction, 90% of the sequenced colonies represented the correct clone, compared with 88.5% for GeneArt® Seamless Cloning and 91.3% for Gibson Assembly. The rest of the colonies represented the original PCR vector template. Colonies with incorrectly assembled DNA fragments were not identified using any of the three cloning methods.

The PIPE cloning method was also applied for swapping heavy chain constant regions within the dual antibody expression vectors. We aimed to exchange the existing Cε region within the pVITRO1-CSPG4-IgE/κ vector without affecting C_K_ and CSPG4-specific V_H_ and V_K_. Using a vector-specific primer pair flanking the existing Cε region, the vector was linearized and subsequently *Dpn*I-treated to destroy the *E. coli*-derived PCR template. Agarose gel electrophoresis analysis of the PCR linearized vector showed no unspecific amplifications (see Supplementary Figure S2 online) and sequencing identified no mutations. A second primer pair with 5′-vector-end overlapping sequences was used for amplification of the incoming Cγ_1_ (see Supplementary Fig. S2 online). The unpurified *Dpn*I-treated linearized vector and Cγ_1_ PCR products were mixed together (v/v) and transformed into competent *E. coli* cells (NEB 10-beta). DNA sequencing of a single colony confirmed the correct assembly of a complete pVITRO1-CSPG4-IgG_1_/κ vector, coding for the expression of chimeric anti-CSPG4 IgG_1_ antibody (see Supplementary Fig. S3 online). Similarly, vectors coding for the expression of Phl p 7 allergen specific fully human IgG_1_, IgG_2_, IgG_3_, IgG_4_, IgA_1_ and IgA_2_ isotypes were generated, demonstrating the system's simplicity to exchange constant-region antibody domains allowing cloning of different antibody isotypes with the same specificity. Furthermore, to investigate the frequency of mutations in the vectors generated by PIPE cloning, we have sequenced the entire vectors. Sequencing results confirmed no mutations were present in the vectors after swapping variable or constant regions.

The overall time required for swapping variable or constant regions within the antibody expression vectors took just a few hours and the individual steps are outlined in Table 1. It is important to mention that any competent *E. coli* cells can be used for the PIPE antibody cloning; however the use of highly competent cells increases the cloning efficiency. Incubating the cloning mixtures for 1 hour at room temperature or overnight at 16°C also improves cloning efficiency.

### Recombinant Antibody Production

**Figure 3:**
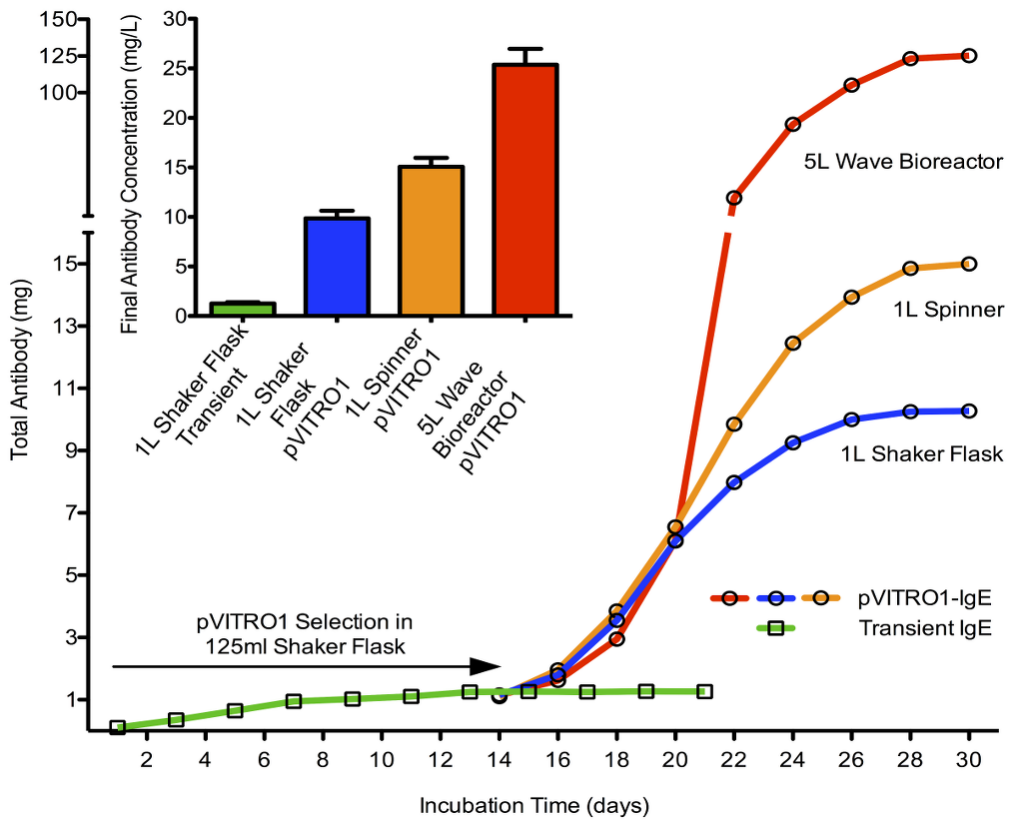
Large-scale production of recombinant antibodies. ELISA based comparison of antibody expression yields, at different time points (main line graph) and final antibody concentration (bar chart), from two HEK 293-F cell cultures transfected with either two transient monocistronic vectors (in squares) or pVITRO1 bicistronic expression vector (in circles) encoding an anti-CSPG4 IgE antibody. Transiently transfected cell cultures (in green) lacking a selection marker terminate within 3 weeks post-transfection. pVITRO1 transfected low volume cell cultures, undergo a two-week hygromycin B selection and are scaled up to larger volume shaker flasks (in blue), spinners (in orange) or bioreactors (in red). The antibody concentration in mg/L was determined by reference to a standard curve and the results represent the mean of triplicate readings ± SD.

Having successfully adapted and optimized the PIPE cloning method for exchange of variable- or constant-region domains within the dual antibody expression cassette in pVITRO1, we aimed to develop a large-scale antibody production for subsequent use in animal model studies. The FreeStyle^TM^ 293-F cell line, a suspension cell line adapted to grow in serum-free conditions, was used for antibody production due to its easy scalability and subsequent purification. Cells were transfected with pVITRO1-CSPG4-IgE/κ and hygromycin B-selected cells expressing anti-CSPG4 IgE were expanded into a 1 L shaker flask, 1 L spinner bottle or 5 L WAVE bioreactor (Figure 3). To compare the final expression yields, 293-F cells were also transfected with transient monocistronic vectors coding for the same antibody. The transient antibody expression system produced a maximal 1 mg/L expression yield by day 13 without significant change until day 21 when the transiently transfected cells died. In contrast, after 2 weeks of hygromycin B selection, the pVITRO1-CSPG4-IgE/κ transfected cells were expanded into a 1 L shaker flask, 1 L spinner and 5 L WAVE bioreactor and produced 10 mg/L, 15 mg/L and 25 mg/L antibody expression levels respectively, 30 days post-transfection. Using a 5 L WAVE bioreactor, a total of 125 mg of antibody was produced within 30 days.

### Antibody Characterization

**Figure 4:**
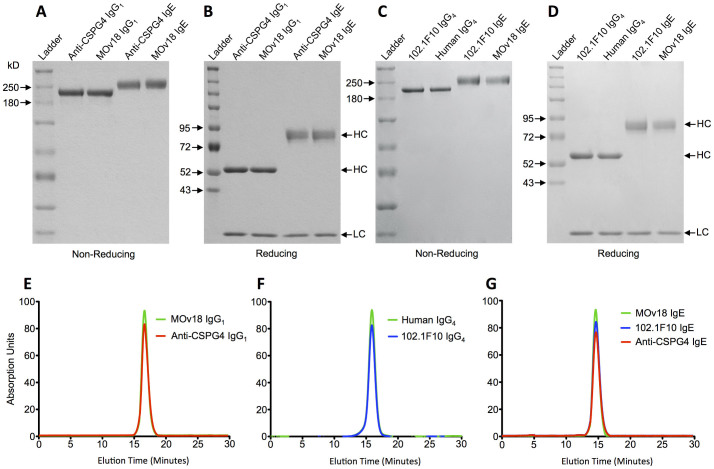
Purified recombinant monoclonal antibodies are complete, monodisperse molecules. (A–D) SDS-PAGE analysis of recombinantly expressed anti-CSPG4 IgG_1_ and IgE antibodies under non-reducing (A) and reducing conditions (B), and 102.1F10 Phl p 7 specific IgE and IgG_4_ antibodies under non-reducing (C) and reducing conditions (D), alongside with isotype controls MOv18 IgE and human myeloma IgG_4_ antibodies respectively, and Spectra^TM^ Multicolor High Range Protein Ladder (Fermentas), visualized by Coomassie blue staining. The represented antibody molecular masses are identical to isotype controls and show the secreted antibodies are properly folded and glycosylated. (E–G) Size exclusion chromatography analysis of the elution profile from purified antibodies corresponds to isotype controls and confirms the purified product consists of monodisperse antibodies.

**Figure 5:**
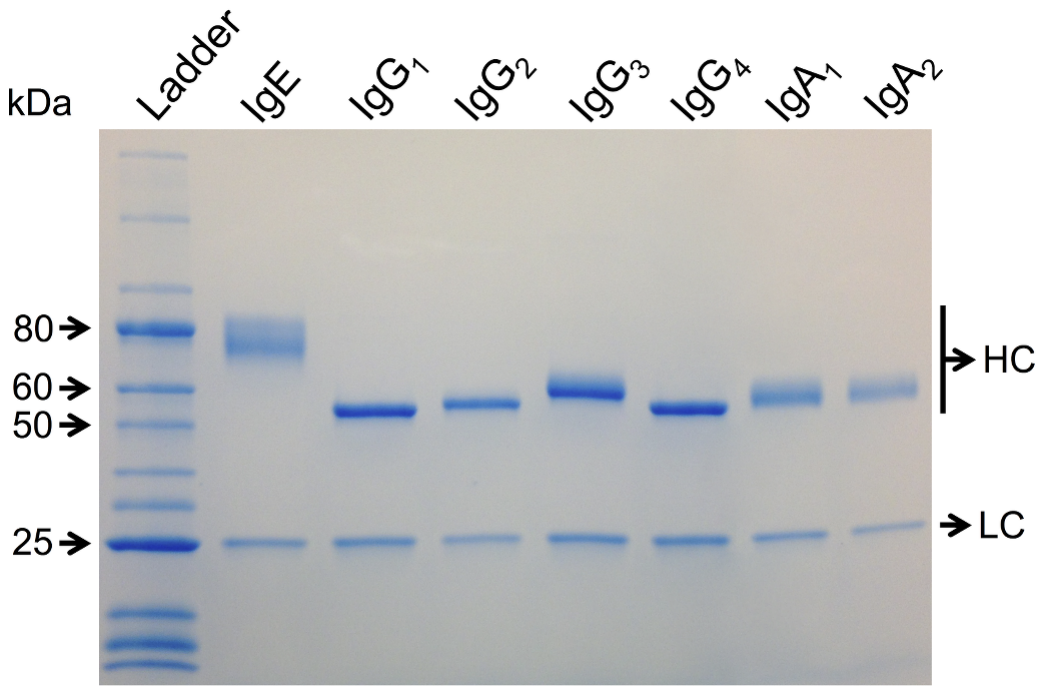
Antibody cloning by PIPE enables parallel characterization of different antibody isotypes. Utilizing the PIPE cloning method seven different human isotype expression vectors were seamlessly generated for production of antibodies in HEK 293-F cells. Purified grass pollen allergen Phl p 7 specific 102.1F10 isotypes were analyzed by SDS-PAGE under reducing conditions, alongside with Protein Ladder (10-250 kDa) (NEB), and visualized by Coomassie blue staining (HC, heavy chain, LC, light chain).

Purity and apparent molecular weight of purified antibodies was assessed by SDS-PAGE analysis. Under non-reducing conditions, apparent molecular sizes were found to be between 180-250 kDa for the heterotetrameric IgG_1_/IgG_4_ antibodies and 250 kDa for the heterotetrameric IgE antibody (Figure 4a,c). Under reducing conditions, protein bands corresponding to the heavy (52–72 kDa) and light chains were identified (Figure 4b,d). Free light or heavy chain was not detected, suggesting that the antibody chains are assembled into whole antibody molecules. The molecular sizes corresponding to the heavy and light chains suggest the secreted antibodies are properly folded and glycosylated^29^,^30^. Similar results were obtained when assessing the IgG_2_, IgG_3_, IgA_1_ and IgA_2_ isotypes under reducing conditions, with protein bands corresponding to the heavy chains (50–60 kDa) and light chains at 25 kDa (Figure 5).

Size-exclusion chromatography was used for biophysical analysis of the purified anti-CSPG4 IgE/IgG_1_, 102.1F10 IgE/IgG_4_ and isotype controls, as described previously^33^. MOv18 IgE/IgG_1_, antibodies directed against the ovarian cancer antigen folate receptor alpha^34^ and a commercially available human myeloma IgG_4_ (Millipore), respectively, were used as isotype controls. The size-exclusion chromatography analysis showed no aggregation and confirmed the purified products consisted of monodisperse antibodies (Figure 4e–g).

### Antibody Functionality

**Figure 6:**
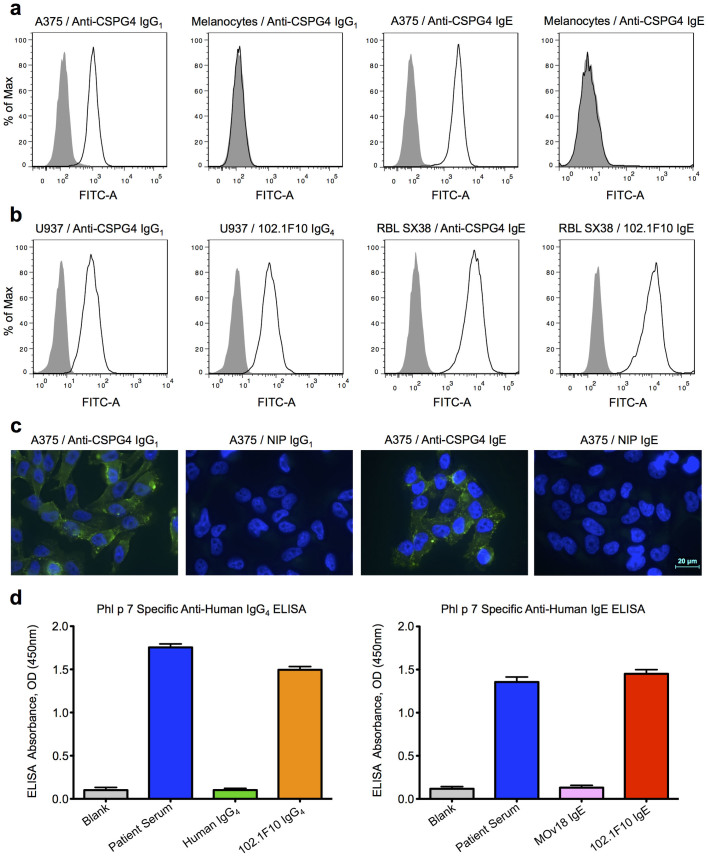
Recombinantly expressed monoclonal antibodies are immunoreactive and functional. (A–B) Flow cytometric histograms showing fluorescent intensity of cells incubated with purified IgG/IgE antibodies plus secondary FITC-labelled goat anti-human IgG/IgE (open) and cells with secondary antibody only (filled) against the number of cells. (A) Flow cytometric assessment of anti-CSPG4 IgG_1_/IgE antibodies shows specific binding to native CSPG4 antigen present on the cell surface of A375 melanoma cells and no binding above background to primary melanocytes. (B) Fc regions of anti-CSPG4 IgG_1_ and 102.1F10 IgG_4_ isotypes demonstrate effector-binding to U937 monocytic cell line, expressing human Fcγ receptors. The IgE antibody isotypes also bind similarly to RBL SX38 mast cells, expressing human FcεR1 receptor. (C) Immunofluorescence staining of A375 cells confirms specific binding of anti-CSPG4 IgG_1_/IgE and no background binding with isotype control hapten specific NIP-IgG_1_/IgE detected by goat anti-human IgG/IgE-FITC. (D) Grass pollen allergen specificity is confirmed by anti-human sandwich ELISA, showing specific binding of recombinant 102.1F10 IgG_4_/IgE and original patient serum to the ELISA plate-bound Phl p 7 allergen and no binding above background with unspecific human myeloma IgG_4_ and MOv18 IgE isotype controls.

The functional characteristics of recombinantly expressed and purified antibodies were assessed by flow cytometric analysis. Both anti-CSPG4 IgE and IgG_1_ antibodies bound to CSPG4^+^ A375 human melanoma cells, but not to CSPG4^−^ primary human melanocytes (Figure 6a). The Fc receptor-binding activities of the antibodies were analysed using the human monocytic cell line U937, which expresses Fcγ receptors at medium densities, or to RBL SX38 rat basophilic leukaemia cells, which express both human and rat high affinity IgE receptor FcεRI^35^. IgE antibodies bound to human FcεRI receptor, expressed on RBL SX38 cells, and IgG_1_/IgG_4_ antibodies bound to Fcγ receptors on the surface of U937 cells (Figure 6b).

Binding of anti-CSPG4 IgE and IgG_1_ antibodies to the surface of the A375 tumour cells was also confirmed by immunofluorescence microscopy, while hapten specific isotype control, NIP-IgE and IgG_1_ antibodies did not show binding above background levels (Figure 6c). Therefore, both flow cytometric and immunofluorescence analyses of anti-CSPG4 IgE and IgG_1_ confirmed that the antibodies recognized and bound their expected tumour target-expressing cells specifically.

Allergen specificity of 102.1F10 IgE/IgG_4_ antibodies was confirmed by means of a sandwich ELISA. The Phl p 7-specific antibodies bound to the allergen coated onto plates at levels comparable to those measured with serum from a patient diagnosed with grass pollen allergy, while the isotype controls showed no binding above background levels (Figure 6d).

## Discussion

Over the last three decades, recombinant mAbs have become a key tool for basic research, diagnosis and treatment of human diseases. The increasing demand for therapeutic antibodies has resulted in a significant improvement in antibody production systems, allowing biopharmaceutical communities to reach grams per liter expression levels. However, new efficient and cost-effective cloning and expression platforms ensuring consistent antibody production are highly desirable to facilitate research using recombinant antibodies in academic settings.

An ideal antibody cloning method would require the minimum costs of labor and reagents and deliver high success rate of correctly assembled expression vectors. Conventional methods for antibody cloning utilize restriction enzymes, ligases and DNA purification kits, which is a labor-intensive procedure introducing additional costs. The exponential growth in the field of molecular cloning in recent years has yielded to the development and availability of efficient and time-effective cloning products such as Gibson Assembly (NEB), GeneArt® Seamless Cloning (Life Technologies) and Gateway® Cloning (Invitrogen). Although these products allow the assembly of multiple DNA fragments in just a few hours, they add an additional cost to the antibody cloning procedure. To overcome this limitation, we have used the Polymerase Incomplete Primer Extension (PIPE) method^32^ to construct a dual antibody expression cassette in the pVITRO1 mammalian expression vector. The PIPE method relies on the inefficiency of the amplification process in the final cycles of a PCR reaction, possibly due to the decreasing availability of dNTPs. This incomplete 5′-3′ primer extension results in a mixture of PCR products with variably single-stranded 5′-ends^36^. These can be used as annealing junctions for PCR products sharing homologous single-stranded 5′-ends, and nicks and gaps are repaired *in vivo* after transformation^32^. In our experiments, antibody cloning by PIPE delivered similar success rate of correctly assembled vectors compared to the commercially available cloning kits (see Supplementary Table S1 online). Adopting the PIPE cloning technique, combined with a single vector system, which simultaneously accommodates antibody heavy and light chains, we developed an efficient and cost-effective method for antibody cloning and production based on previously established disparate technologies.

Here, cloning the heavy and light antibody chains is conducted in just a few hours, and in principle, this could be applied for the generation of expression vectors of antibodies of any species (see Supplementary Table S2 online) and isotype with any desired specificity, given the availability of DNAs encoding the heavy- and light-chain V and C regions. When combined with the downstream transfection of pVITRO1 expression vectors into Human Embryonic Kidney (HEK) 293-F cells, followed by hygromycin B selection, this strategy can yield reproducible generation of tens of milligram quantities of functional recombinant mAb in less than four weeks from cloning of the V- and C-region DNA, through to harvesting of selected cell supernatants. Recombinant mAbs have been produced in HEK 293-F cells^25^,^26^,^27^,^37^,^38^ and although cloning the dual antibody cassette into pVITRO1 for expression in these cells has facilitated appreciable expression yields (Figure 3), the cassette can be transferred to any compatible expression vector consisting of two transcription units for use in alternative systems, if required.

Using this method, we have generated vectors coding for the expression of antibodies of different isotypes directed against two different antigens (CSPG4, Phl p 7). In principle, the method could be advantageous for the functional analysis of V gene libraries derived from isolated mRNA from single B cells or by phage display, since an extensive panel of recombinant antibodies may be generated in a relatively short period of time. Additionally, C-region exchange to generate antibodies of different isotypes could enable the direct comparison of different antibody effector functions and also of inter-species differences. Here we have produced fully human IgG_1_, IgG_2_, IgG_3_, IgG_4_, IgE, IgA_1_ and IgA_2_ isotypes (Figure 5) with the same specificity to allergen Phl p 7 and in the field of allergen immunotherapy it may be desirable to compare the properties of different antibody isotypes in order to select the most efficacious antibody treatment. In another example, different isotypes specific for the tumor-associated antigen CSPG4 can be produced to elucidate their potential antitumor effects against solid cancers.

In summary, we report a platform entailing antibody cloning in a single mammalian expression vector and scalable production process that can deliver appreciable protein yields to facilitate a variety of pre-clinical studies and future clinical applications.

## Methods

### Construction of Dual Antibody Expression Vectors by PIPE

pVITRO1 (InvivoGen) vector-specific oligonucleotides (desalted, Sigma) MCS2_F and MCS2_R (Table 2) were used for PCR vector linearization at multiple cloning site 2 (MCS2). A PCR reaction was set up containing 0.5 μM each of these primers, 25 μl 2× Phusion^TM^ Flash High-Fidelity PCR Master Mix (Finnzymes), 10 ng of pVITRO1 vector template and MilliQ water to 50 μl. The reaction was treated as follows: initial denaturation for 30 seconds at 98°C, then 30 cycles of denaturation for 10 seconds at 98°C, annealing for 15 seconds at 60°C and extension for 1 minute 50 seconds at 72°C, followed by a cool down to 4°C. The linearized vector was subsequently treated with *Dpn*I (New England Biolabs) according to the manufacturer's instructions. In parallel, a human ε heavy chain expression cassette (V_H_1-02 secretary leader, V_H_ and Cε) was amplified from a monocistronic vector pIgε^39^ using primers HC_F and HC_R (Table 2), which included 5′-ends complementary to the MCS2 of pVITRO1 and 3′-ends complementary to the V_H_1-02 secretary leader and Cε terminus respectively. The heavy chain PCR reaction was treated exactly as above, except the extension step was reduced to 20 seconds at 72°C. Unpurified PCR products were mixed 1:1 (v/v) and 2 μl of the mixture were transformed into chemically competent *E. coli* cells (High Efficiency NEB 10-beta). Transformation was performed according to the manufacturer's instructions, except the culture was not diluted in SOC media after incubation at 37°C and 200 μl were plated onto Fast-Media® Hygro Agar LB petri dishes (InvivoGen). In a subsequent PIPE cloning reaction, a sequence verified clone pVITRO1-IgE (contacting ε heavy chain expression cassette at MCS2) was PCR linearized at MCS1 by vector-specific primers MCS1_F and MCS1_R (Table 2) with extension step for 2 minutes at 72°C, followed by a *Dpn*I treatment. A human kappa light chain expression cassette (VκA26 secretary leader, Vκ and Cκ) was amplified from a monocistronic vector pIgκ^39^ using primers KC_F and KC_R (Table 2), in which the 5′-ends are complementary to the MCS1 of pVITRO1 and 3′-ends complementary to the VκA26 secretary leader and Cκ terminus respectively. Similarly, a human lambda light chain expression cassette (Vλ 8a secretary leader, Vλ and Cλ) was amplified from a monocistronic pIgλ vector^12^ using primers LC_F and LC_R (Table 2). Light chain PCR reactions included an extension step for 10 seconds at 72°C. Unpurified *Dpn*I-treated linearized vector was mixed 1:1 (v/v) with unpurified kappa or lambda light chain PCR product and 2 μl of the mixture were transformed into competent *E. coli* cells as described above.

### Swapping Antibody Variable Regions by PIPE

The dual antibody expression vectors were used to swap antibody variable regions by PIPE cloning. pVITRO1-IgE/κvector was linearized by two sets of vector-specific primer pairs flanking the existent V_K_ and V_H_; Linear_Kfwd with Linear_Hrev and Linear_Hfwd with Linear_Krev (Table 2), in two independent PCR reactions. The PCR reactions were treated exactly as above, except the extension step was carried out for 55 seconds at 72°C, followed by a *Dpn*I treatment of the two resulting PCR vector fragment products. Simultaneously, CSPG4-specific V_H_ and V_K_ were PCR amplified from previously cloned monocistronic pIgε-CSPG4 and pIgκ-CSPG4 vectors, bearing previously published CSPG4 specific variable regions^40^. Two sets of primer pairs; CSPG4H_Fwd with CSPG4H_Rev and CSPG4K_Fwd with CSPG4K_Rev (Table 2), were used in two independent PCR reactions with extension step for 5 seconds at 72°C. In the first primer pair CSPG4H_Fwd and CSPG4H_Rev, the 5′-ends are complementary to the V_H_1-02 secretary leader terminus and start of Cε respectively, while 3′-ends are CSPG4-V_H_ specific. In the second primer pair CSPG4K_Fwd and CSPG4K_Rev, the 5′-ends are complementary to the Vκ A26 secretary leader terminus and start of Cκ respectively, while 3′-ends are CSPG4-V_K_ specific. The *Dpn*I-treated unpurified vector fragments were mixed with the unpurified CSPG4 specific V_H_ and V_K_ in 1:1:1:1 (v/v) ratio and 2 μl from the mixture transformed into competent *E. coli* cells as described above, assembling a pVITRO1-CSPG4-IgE/κ vector. Similarly, pVITRO1-IgE/λ vector was linearized by two sets of vector-specific primer pairs; Linear_Lfwd with Linear_Hrev and Linear_Hfwd with Linear_Lrev (Table 2), and Phl p 7 specific (102.1F10) V_H_ and V_K_ were PCR amplified from monocistronic vectors^12^ by two sets of primer pairs; 102.1F10H_Fwd with 102.1F10H_Rev and 102.1F10L_Fwd with 102.1F10L_Rev, for the assembling reaction of the pVITRO1-102.1F10-IgE/λ vector.

To compare the cloning efficiency between different DNA assembly methods, vector pVITRO1-CSPG4-IgE/κ was cloned by PIPE, Gibson Assembly (New England Biolabs) and GeneArt® Seamless Cloning (Life Technologies) method. The two vector fragments, amplified from pVITRO1-IgE/κ vector template, were mixed with vector fragment terminal end-homologous CSPG4 specific V_H_ and V_K_ in a PIPE cloning reaction as previously described, while Gibson Assembly and GeneArt® Seamless Cloning kits were used according to the manufacturer's specifications.

### Swapping Antibody Constant Regions by PIPE

The PIPE cloning method was also used to swap constant regions within the dual antibody expression vectors. pVITRO1-CSPG4-IgE/κ vector was linearized by primer pair pAn_Fwd and CSPG4-VH_Rev (Table 2), flanking the Cε region. The PCR reaction was treated exactly as above, except the extension step for 2 minutes at 72°C, followed by *Dpn*I treatment. Simultaneously, a human Cγ_1_ region was amplified from a monocistronic pIgγ1 vector^39^ using primer pair Cg1_Fwd and Cg1_Rev (Table 2), in which the 5′-ends are complementary to the CSPG4 V_H_ terminus and vector sequence downstream of Cε respectively, while the 3′-ends are Cγ_1_ specific. The Cγ_1_ region PCR had an extension step for 10 seconds at 72°C. Unpurified *Dpn*I-treated linearized vector was mixed in 1:1 (v/v) ratio with unpurified Cγ_1_ region PCR product and transformed into competent *E. coli* cells as described above, assembling pVITRO1-CSPG4-IgG_1_/κ vector. pVITRO1-102.1F10-IgE/λ vector was linearized by primer pair pAn_Fwd1 and 102.1F10-VH_Rev (Table 2), flanking the Cε region, and human Cγ_4_ was amplified from a pIgγ4 vector^41^ using primer pair Cg4_Fwd and Cg4_Rev (Table 2). Unpurified PCR products were mixed and transformed into competent *E. coli* cells as described above, assembling pVITRO1-102.1F10-IgG_4_/λ vector. Similarly, the linearized pVITRO1-102.1F10-IgE/λ vector was mixed with vector-end homologous PCR amplified human Cγ_1_, Cγ_2_, Cγ_3_, Cα_1_ and Cα_2_ to generate Phl p 7 specific human IgG_1_, IgG_2_, IgG_3_, IgA_1_ and IgA_2_ expression vectors, which have been deposited with Addgene (http://www.addgene.org/Andrew_Beavil).

### Cell Lines

Human monocytic cell line U937 (CRL-1593.2, ATCC)^42^, expressing Fcγ receptors at medium densities, was grown in RPMI 1640 medium supplemented with 10% FCS and 2 mM L-glutamine. The human melanoma cell line A375 (CRL-1619, ATCC)^43^, naturally expressing the High Molecular Weight Melanoma Associated Antigen (HMW-MAA, or CSPG4) was maintained in Dulbecco's Modified Eagles Media (DMEM) supplemented with 10% FCS and 2 mM L-glutamine. Primary human epidermal melanocytes (PCS-200-012, ATCC) were grownin Dermal Cell Basal Medium (PCS-200-030, ATCC) and supplemented with the Melanocyte Growth Kit (PCS-200-041, ATCC). The RBL SX-38 rat basophilic leukaemia cell line^35^, which stably expresses the human tetrameric (αβγ2) high-affinity IgE receptor (FcεRI) was a kind gift from Prof. J-P. Kinet (Harvard University, Boston, MA) and was grown in DMEM supplemented with 10% FCS, 500 μg/mL Geneticin and 2 mM L-glutamine. Suspension serum-free adapted FreeStyle^TM^ 293-F cells (R790-07, Life Technologies)^44^ were cultured in FreeStyle^TM^ 293 Expression Medium. All cell lines were supplemented with penicillin (5000 U/mL) and streptomycin (100 μg/mL) and maintained in a 5% CO_2_ humidified incubator at 37°C.

### Recombinant Antibody Production

Suspension and serum-free adapted FreeStyle^TM^293-F cells (Life Technologies) cultured in FreeStyle^TM^293 Expression Medium (Life Technologies) were grown in 125 mL sterile Erlenmeyer flasks with vented caps (Sigma) at densities between 1 × 10^5^–5 × 10^5^ viable cells/mL, rotating at 135 rpm on an orbital shaker platform. On the day of transfection, each 125 mL flask containing 30 mL of cells at 1 × 10^6^ viable cells/mL was transfected with either two transient monocistronic vectors (pIgε-CSPG4 and pIgκ-CSPG4) or dual antibody expression vectors (pVITRO1-CSPG4-IgE/κ, pVITRO1-102.1F10-IgE/λ, pVITRO1-CSPG4-IgG_1_/κ and pVITRO1-102.1F10-IgG_4_/λ) using FreeStyle^TM^MAX transfection reagent (Life Technologies) according to the manufacturer's instructions. 24 h post-transfection, hygromycin B (50 μg/mL) was added to cells transfected with pVITRO1 vectors. Cells were then maintained under selection for 2 weeks at densities between 2 × 10^5^ - 5 × 10^5^ viable cells/mL, followed by expansion into a 1 L shaker flask (Sigma), 1 L spinner bottle (Sigma) or 5 L WAVE bioreactor (GE Healthcare). To compare expression yields between cells transfected with two transient monocistronic vectors (pIgε-CSPG4 and pIgκ-CSPG4) and a dual antibody expression vector (pVITRO1-CSPG4-IgE/κ), samples were collected every 48 h and IgE expression levels were determined by anti-human IgE ELISA as described previously^45^. Cultured supernatants were harvested after 16 days, centrifuged at 1000 × g for 15 minutes, passed over 0.45 μm filters (Sartorius) and stored at 4°C with 0.1% sodium azide (Sigma) until use.

### Purification of Recombinant Antibodies

Chimeric anti-CSPG4 IgG_1_ and human 102.1F10 IgG_1–4_ were purified by affinity chromatography with a 5 mL HiTrap Protein-G HP column (GE Healthcare) using an ÄKTA Prime system (GE Healthcare) and 0.2 μm filtered buffers. The column was equilibrated with 10 Column Volumes (CV) of phosphate buffered saline (PBS) washing buffer (pH 7.0) and the supernatant loaded at a flow rate of 2 mL/min, followed by 10 CV of washing buffer. The antibody was eluted with 0.2 M Glycine buffer (pH 2.3) and 2.5 mL fractions were collected into tubes containing 0.5 mL 1 M Tris–HCl pH 8.6 for neutralization.

Chimeric anti-CSPG4 IgE and human 102.1F10 IgE/IgA_1_/IgA_2_ were purified by mixed-mode chromatography with a 5 mL pre-packed MEP HyperCel^TM^ column (Pall Corporation) using an ÄKTA Prime system as described previously^46^. Briefly, the column was equilibrated with 10 CV of washing buffer (PBS, pH 7.0) and the supernatant loaded at a flow rate of 2 mL/min, followed by 20 CV washing buffer and 20 CV sterile water. Step-elution with a series of 5 CV of 50 mM sodium acetate buffers with incrementally decreasing pH values (5.6–3.0) was used to elute the IgE antibodies at pH 5.2 and the IgA antibodies at pH 4.9. The column was cleaned with 1 M NaOH for 60 minutes and re-equilibrated with 20 CV washing buffer between runs.

### SDS-Polyacrylamide Gel Electrophoresis

Purity and apparent molecular weight of purified antibodies was assessed by SDS-PAGE analysis. 1 μg of each antibody was subjected to a gradient 5–12% polyacrylamide gel under non-reducing and 5–20% gel under reducing conditions. For reduction, 10% β-mercaptoethanol was added to the denaturating sample buffer and all samples were incubated for 2 minutes at 90°C. Spectra^TM^ Multicolor High Range Protein Ladder (Fermentas) or Protein Ladder (10–250 kDa) (New England Labs) were used to assess the approximate size of proteins visualized by Coomassie blue staining.

### Size exclusion chromatography

Purified antibodies were analysed by size exclusion chromatography as previously described^33^. Briefly, gel filtration was performed on a Gilson HPLC system using a Superdex™ 200 10/300 GL column (GE Healthcare), suitable for purifying proteins between 10–300 kDa, at a flow rate of 0.75 mL/min in PBS (pH 7.0, 0.2 μm filtered).

### Flow Cytometry

Flow cytometry experiments were performed to assess antigen binding of purified anti-CSPG4 IgE and IgG_1_ to CSPG4^+^A375 melanoma cells (ATCC) and CSPG4^−^primary human melanocytes (ATCC), Fcγ receptor binding of anti-CSPG4 IgG_1_ and 102.1F10 IgG_4_ to U937 cells (ATCC) expressing Fcγ receptors, and Fcε receptor binding of anti-CSPG4 IgE and 102.1F10 IgE to RBL-SX38 cells^35^ expressing the human FcεRI receptor. Cells were incubated with 0.4 μg/mL purified antibodies for 30 minutes at 4°C, followed by two washes in PBS supplemented with 5% normal goat serum (FACS buffer). Cells were then incubated with either 10 μg/mL goat anti-human IgE-FITC (Vector Labs) or goat anti-human IgG-FITC (Jackson ImmunoResearch) for 30 minutes at 4°C and washed with FACS buffer prior to acquisition and analysis on a FACS Canto^TM^ flow cytometer (BD Biosciences).

### Immunofluoresence staining of cells grown on glass chamber slides

A375 melanoma cells (ATCC) were seeded in LabTek glass chamber slides (Nunc) at a density of 2 × 10^4^ cells per well. Cells were washed in PBS and fixed in 4% formaldehyde PBS for 15 minutes and then incubated with PBS-T, 10% goat serum, 0.05% Tween for 25 minutes at room temperature. Anti-CSPG4 IgG_1_/IgE or control NIP antibodies were incubated for 45 minutes with A375 cells at a concentration of 10 μg/mL. Cell-bound antibodies were detected with a secondary goat anti-human IgG-FITC (Jackson ImmunoResearch) or goat anti-human IgE antibody conjugated to FITC (Vector Labs). Nuclei were stained in Hoechst dye for 3 minutes. All washing steps used PBS, except the final wash with dH_2_0. Cells were then mounted in Mowiol (Sigma) mounting medium. Fluorescence microscopy was performed on a Zeiss Axiovert Z.1 (40× objective) upright microscope. AxioCamMR3 and AxioVision Software (Carl Zeiss) was used for acquisition and analysis.

### Phl p 7 specific anti-human ELISA

ELISA plates (Maxisorp, Nunc) were coated with 5 μg/mL of recombinant Phl p 7 (MRC/Asthma UK Protein Production Facility, King's College London, UK). Plates were blocked with PBS/1% BSA and washed throughout with PBS/0.05% Tween-20. 1 μg/mL purified 102.1F10 IgG_4_/IgE, negative isotype controls human myeloma IgG_4_ (Calbiochem), MOv18 IgE^34^ and positive control Phl p 7 reactive patient serum^12^ were incubated in triplicate for 2 hours at room temperature, followed by incubation with isotype specific antibodies. IgG_4_ antibodies were detected with mouse anti-human IgG_4_ biotin-conjugated antibody (clone G17-4, BD Biosciences), incubated at 1 μg/mL, followed by streptavidin–horseradish peroxidase (R&D Systems). IgE antibodies were detected with polyclonal goat anti-human IgE (Sigma-Aldrich) directly conjugated with horseradish peroxidase using the manufacturer's recommended dilutions. ELISA plates were developed using TMB (R&D Systems) by measuring absorbance at 450 nm.

### Ethics Statement

Human serum was obtained with written, informed consent and the London and City Research Ethics Committee approved the study.

## Author Contributions

T.S.D. and A.J.B. designed the experiments and analyzed the data; T.S.D. developed the cloning system and cloned, expressed, purified and characterized the antibodies; P.K. carried out the immunofluorescence microscopy; T.S.D., A.E.G. and D.H.J. performed the flow cytometry; H.B. helped optimise the Phl p 7 specific ELISA; T.S.D. prepared figures assisted by L.K.J., H.J.B., R.B. and M.O.P.; S.N.K. designed and supervised microscopy and flow cytometry experiments; H.J.G. designed and supervised ELISA experiments; H.J.G. and S.N.K. commented on the manuscript; T.S.D. wrote the paper; All authors reviewed the manuscript.

## Supplementary Material

Supplementary Information Supplementary Information

## Figures and Tables

**Table 1:** PIPE Cloning Timing. PIPE cloning reaction steps performed within a few hours

Step	Swapping V_κ/λ_ & V_H_	Swapping C_H_
PCR (Phusion-Flash)	60 min	90 min
*Dpn*I Digest	15 min	15 min
Transformation	105 min	105 min
**Total**	**3 hours**	**3.5 hours**

**Table 2:** Primers. Desalted Oligonucleotides (Sigma) used for PIPE cloning

Primer	5′ – 3′ Sequence
**MCS2_F**	taggtgttgtgaaaaccaccgctaattcaaagcaaccggt
**MCS2_R**	catcaatgtatcttatcatgtctggccagctagctgtaca
**MCS1_F**	aggtgttgtgaaagccaccgctaattcaaagcaatccgga
**MCS1_R**	tgtgtcattggggaaacctgctcctaggcgtacgggatcc
**HC_F**	gctaattcaaagcaaccggtatggactggacctggaggat
**HC_R**	tctggccagctagctgtacatcatttaccgggatttacag
**KC_F**	ctaattcaaagcaatccggaatgttgccatcacaactcat
**KC_R**	ctcctaggcgtacgggatccctaacactctcccctgttga
**LC_F**	caccgctaattcaaagcaatatggcctggatgatgcttct
**LC_R**	ttggggaaacctgctcctagctatgaacattctgtagggg
**Linear_Hfwd**	gctagcacacagagcccatccgtcttccccttgacccgct
**Linear_Hrev**	ggagtgcgcgcctgtggcggccgccaccaagaagaggatc
**Linear_Kfwd**	cgtacggtggcggcgccatctgtcttcatcttcccgccat
**Linear_Krev**	accgcggctagctggaacccagagcagcagaaacccaatg
**Linear_Lfwd**	ctaggtcagcccaaggcggcgccctcggtcactctgttcc
**Linear_Lrev**	agagtcgactccggatccataagcaaggagtccgaggaga
**CSPG4H_Fwd**	ccgccacaggcgcgcactcccaagtcaaactgcagcagag
**CSPG4H_Rev**	gatgggctctgtgtgctagcgctgctgacagtcacggtgg
**CSPG4K_Fwd**	gggttccagctagccgcggtgacatcgagctgacccagag
**CSPG4K_Rev**	gatggcgccgccaccgtacgcttgatttccagcttggtgc
**102.1F10H_Fwd**	ccgccacaggcgcgcactcccaggtccagcttgtgcagtc
**102.1F10H_Rev**	gatgggctctgtgtgctagctgaggagacggtgaccaggg
**102.1F10L_Fwd**	atggatccggagtcgactctcagtctgccctgactcagcc
**102.1F10L_Rev**	gccgccttgggctgacctaggacggtcagtttggtcccgc
**pAn_Fwd**	tgtacagctagctggccagacatgataagatacattgatg
**pAn_Fwd1**	ctagctggccagacatgataagatacattgatgagtttgg
**CSPG4-VH_Rev**	gctgctgacagtcacggtggtgccctggccccagtggtcg
**102.1F10-VH_Rev**	tgaggagacggtgaccagggctccctggccccaggagtca
**Cg1_Fwd**	ccaccgtgactgtcagcagcgctagcaccaagggcccatc
**Cg1_Rev**	tctggccagctagctgtacatcatttacccggagacaggg
**Cg4_Fwd**	ccctggtcaccgtctcctcagctagcaccaagggcccatc
**Cg4_Rev**	tatcatgtctggccagctagtcatttacccagagacaggg
