# Supplementary material for: A tool kit for rapid cloning and expression of recombinant antibodies
Source: Sci Rep. 2014 Jul 30;4:5885. doi: 10.1038/srep05885 (PMC4115235; doi:10.1038/srep05885)
Supplement: Supplementary Information [file srep05885-s1.pdf]

A tool kit for rapid cloning and expression of recombinant antibodies

Tihomir S Dodev[1,4], Panagiotis Karagiannis[1,2], Amy E Gilbert[1,2], Debra H Josephs[1,2,3], Holly Bowen[1,4], Louisa K James[4], Heather J Bax[4], Rebecca Beavil[4], Marie O Pang[4], Hannah J Gould[4], Sophia N Karagiannis[1,2] and Andrew J Beavil[4]*

Supplementary Figure S1. Schematic representation of expression vectors

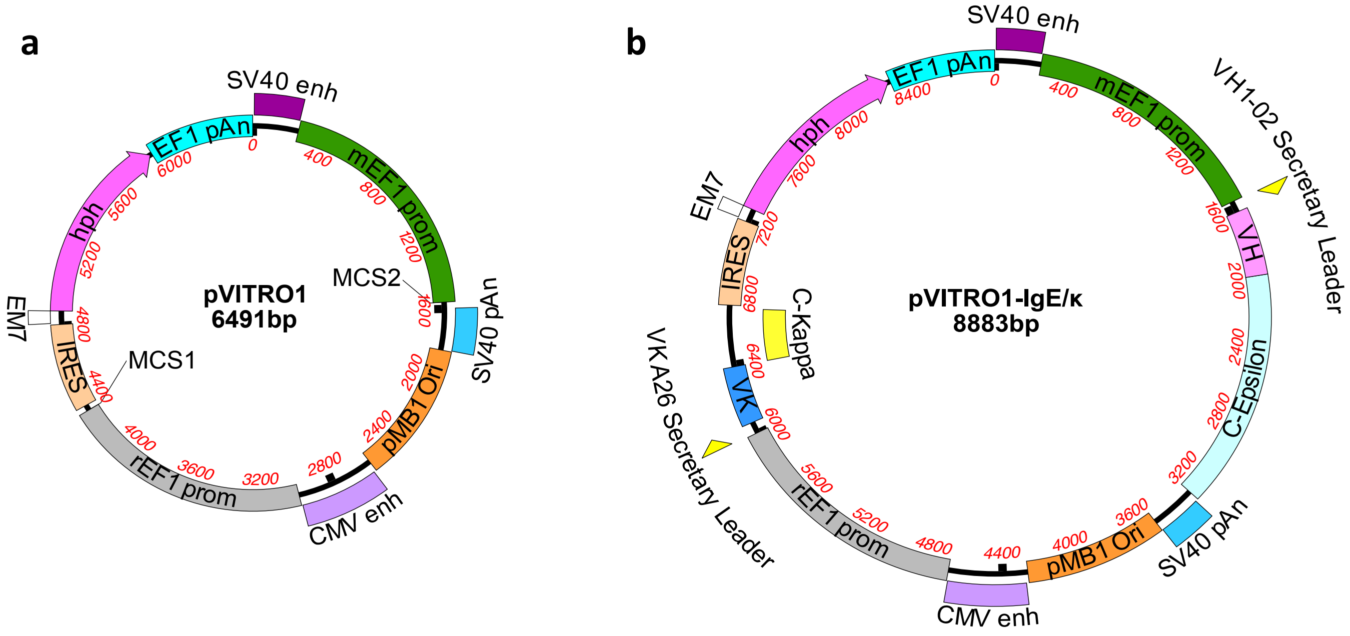

Comments for pVITRO1 6491 nucleotides:

Simian Virus 40 enhancer (SV40 enh):	**8-242** bp
Mouse Elongation Factor 1 alpha promoter (mEF1 prom):	**249-1562** bp
Multiple Cloning Site 2 (MSC2):	**1563-1596** bp
Simian Virus 40 late polyadenylation signal (SV40 pAn):	**1597-1822** bp
Minimal *E. coli* origin of replication (pMB1 Ori):	**1828-2569** bp
Human CMV enhancer:	**2584-2988** bp
Rat Elongation Factor 1 alpha promoter (rEF1 prom):	**2995-4307** bp
Multiple Cloning Site 1 (MSC1):	**4308-4337** bp
Internal Ribosome Entry Site (IRES):	**4338-4806** bp
Bacterial promoter EM7:	**4815-4880** bp
Hygromycin B resistant hph gene:	**4881-5906** bp
EF1 polyadenylation signal:	**5918-6490** bp

Comments for pVITRO1-IgE/κ 8883 nucleotides:

Simian Virus 40 enhancer (SV40 enh):	**8-242** bp
Mouse Elongation Factor 1 alpha promoter (mEF1 prom):	**249-1562** bp
Human VH1-02 Secretary Leader:	**1568-1624** bp
Variable Heavy (VH) region:	**1625-1991** bp
Human Epsilon Constant region (C-Epsilon):	**1992-3277** bp
Simian Virus 40 late polyadenylation signal (SV40 pAn):	**3293-3518** bp
Minimal *E. coli* origin of replication (pMB1 Ori):	**3524-4265** bp
Human CMV enhancer:	**4280-4684** bp
Rat Elongation Factor 1 alpha promoter (rEF1 prom):	**4691-6003** bp
Human VK A26 Secretary Leader:	**6010-6066** bp
Variable Kappa (VK) region:	**6067-6388** bp
Human Kappa Constant region (C-Kappa):	**6389-6711** bp
Internal Ribosome Entry Site (IRES):	**6730-7198** bp
Bacterial promoter EM7:	**7207-7272** bp
Hygromycin B resistant hph gene:	**7272-8298** bp
EF1 polyadenylation signal:	**8310-8882** bp

a) pVITRO1 mammalian expression vector with two multiple cloning sites (MCS), allowing the co-expression of a pair of genes from two different transcription units.

b) b) pVITRO1-IgE/κ antibody expression vector with human epsilon heavy chain expression cassette integrated within MCS2 under the action of SV40 enhancer and human kappa light chain expression cassette within MCS1 under human CMV enhancer.

Supplementary Figure S2. Gel electrophoresis analysis of PIPE amplified DNA products

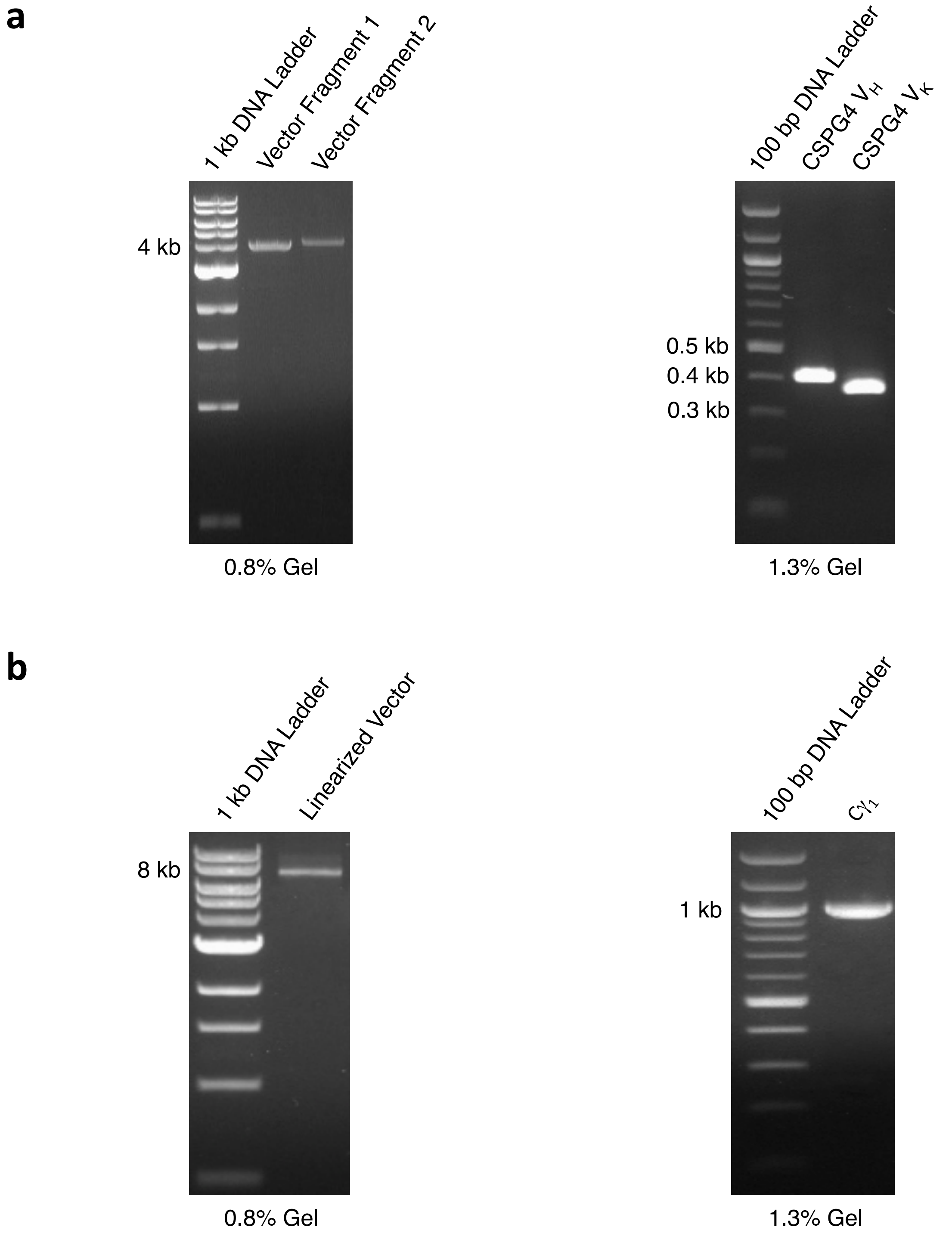

a) Bands representing the two vector fragments (0.8% gel) amplified from vector pVITRO1-IgE/κ by V_H and V_K flanking primer pairs in two independent PCR reactions, and vector fragment terminal end-homologous V_H and V_K (1.3% gel) , alongside with 1 kb and 100 bp DNA ladders respectively.

b) Bands representing the PCR linearized vector pVITRO1-CSPG4-IgE/κ (0.8% gel) by epsilon constant region flanking primer pair, and vector terminal end-homologous human Gamma 1 constant region (1.3% gel), alongside with 1 kb and 100 bp DNA ladders respectively. The electrophoresis analysis shows clear DNA products with no unspecific amplifications.

Supplementary Figure S3. Schematic representation of PIPE cloning strategy for swapping antibody constant regions

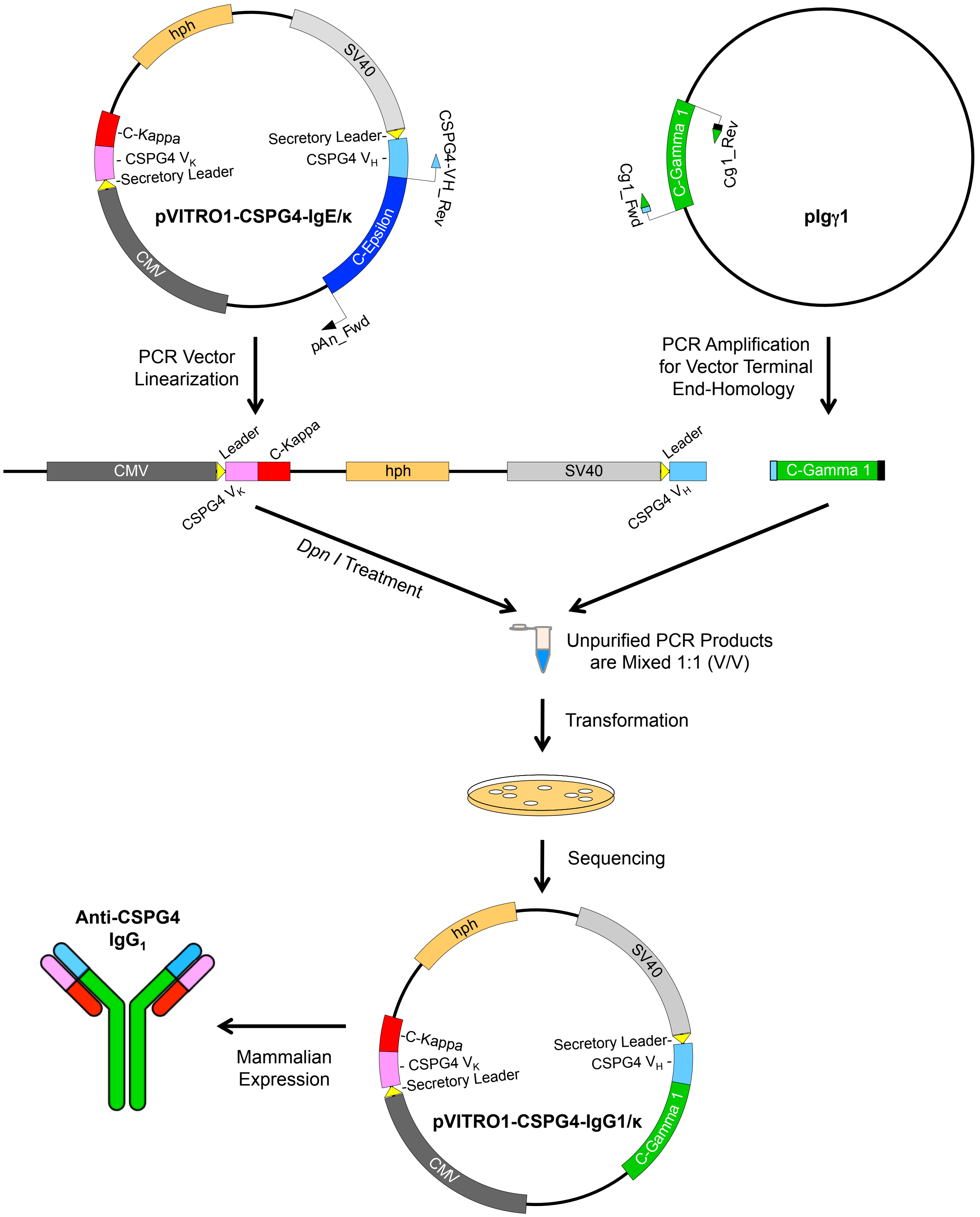

pVITRO1-CSPG4-IgE/κ expression vector is PCR linearized by Epsilon constant region flanking primer pair and subsequently *Dpn*I-treated. Simultaneously, human Gamma 1 constant region is PCR amplified for generation of vector terminal end-homology. The unpurified *Dpn*I-treated linearized vector is mixed 1:1 (v/v) with unpurified Gamma 1 PCR product. The single-stranded DNA fragments anneal directionally across the complementary sequences and nicks and gaps are repaired *in vivo* after transformation, generating pVITRO1-CSPG4-IgG$_1$/κ expression vector.

Supplementary Table S1. Comparison of cloning efficiency of different vector assembly methods.

Single Colonies	GENEART Seamless Cloning and Assembly	Gibson Assembly	Polymerase Incomplete Primer Extension (PIPE)
Sequenced	26	23	20
Positive	23 (88.5%)	21 (91.3%)	18 (90%)
False-Positive	3 (11.5%)	2 (8.7%)	2 (10%)
Negative	0 (0%)	0 (0%)	0 (0%)

Vector pVITRO1-CSPG4-IgE/κ was assembled using GENEART Seamless Cloning and Assembly, Gibson Assembly or Polymerase Incomplete Primer Extension (PIPE). Positive colonies represent the correctly assembled vector, verified by sequencing over the annealing junctions. False-Positive refer to the vector template, used for vector linearization, undigested by the *Dpn*I enzyme. Negative represent colonies, which do not carry pVITRO1 vector.

Supplementary Table S2. Antibody expression vectors

Species	Isotype							
Human	IgE/κ/λ	IgG_1/κ/λ	IgG_2/κ/λ	IgG_3/κ/λ	IgG_4/κ/λ	IgA_1/κ/λ	IgA_2/κ/λ	IgM/κ/λ
Rat	IgE/κ	-	IgG_2b/κ	-	-	-	-	-
Mouse	IgE/λ	-	-	-	-	-	-	-

pVITRO1 antibody expression vectors generated by the PIPE cloning method.
